# Success in Developing Regions: World Records Evolution through a Geopolitical Prism

**DOI:** 10.1371/journal.pone.0007573

**Published:** 2009-10-28

**Authors:** Marion Guillaume, Nour El Helou, Hala Nassif, Geoffroy Berthelot, Stéphane Len, Valérie Thibault, Muriel Tafflet, Laurent Quinquis, François Desgorces, Olivier Hermine, Jean-François Toussaint

**Affiliations:** 1 IRMES, INSEP, Paris, France; 2 Université Paris-Descartes, Paris, France; 3 INSERM unit 970, Paris, France; 4 Service d'hématologie Hôpital Necker and CNRS UMR 8147, Paris, France; 5 CIMS, Hôtel-Dieu, Assistance Publique Hôpitaux de Paris, Paris, France; Universidad Europea de Madrid, Spain

## Abstract

A previous analysis of World Records (WR) has revealed the potential limits of human physiology through athletes' personal commitment. The impact of political factors on sports has only been studied through Olympic medals and results. Here we studied 2876 WR from 63 nations in four summer disciplines. We propose three new indicators and show the impact of historical, geographical and economical factors on the regional WR evolution. The south-eastward path of weighted annual barycenter (*i.e.* the average of country coordinates weighting by the WR number) shows the emergence of East Africa and China in WR archives. Home WR ratio decreased from 79.9% before the second World War to 23.3% in 2008, underlining sports globalization. Annual Cumulative Proportions (ACP, *i.e.* the cumulative sum of the WR annual rate) highlight the regional rates of progression. For all regions, the mean slope of ACP during the Olympic era is 0.0101, with a maximum between 1950 and 1989 (0.0156). For European countries, this indicator reflects major historical events (slowdown for western countries after 1945, slowdown for eastern countries after 1990). Mean North-American ACP slope is 0.0029 over the century with an acceleration between 1950 and 1989 at 0.0046. Russia takes off in 1935 and slows down in 1988 (0.0038). For Eastern Europe, maximal progression is seen between 1970 and 1989 (0.0045). China starts in 1979 with a maximum between 1990 and 2008 (0.0021), while other regions have largely declined (mean ACP slope for all other countries  = 0.0011). A similar trend is observed for the evolution of the 10 best performers. The national analysis of WR reveals a precise and quantifiable link between the sport performances of a country, its historical or geopolitical context, and its steps of development.

## Introduction

Olympic Games (OG) were reintroduced in 1896 in order to promote pacific relations between nations, but many sports competitions in the 20^th^ century favored direct confrontation in a “gourmand” quest of world records (WR). Previous studies have underlined the link between individual physiology and maximal human species performances [1], [2]. Others studies [3], [4] analyzed the Olympic performances of nations through their medals number and showed the effect of historical and geographical factors. However the number of medals obtained over a 4 year period gives less information than a study of quantifiable events obtained on a yearly basis. These allow for a more precise understanding of national strategies and of states involvement in high-level sport competitions. WR are the extreme values of human physiological capabilities. We tested the hypothesis that world records may also reflect the impact of historical or political events (World Wars, Great Depression, Cold War, Cultural Revolution, USSR end) on human phenotype as assessed by sport performances.

## Materials and Methods

### Data

Data consist of 2876 WR and 4672 Olympic medals from 4 quantifiable disciplines of summer OG: Track and Field, Swimming, Weightlifting and Cycling. Data were gathered from 1896 to 2008 (modern Olympic era) [5]–[10]. For each WR and Olympic medal, citizenship of the athlete and location of the event are collected. Forty-two host nations generated “Home WR” and 16 generated Home Olympic Medals (*i.e.* 16 OG organizers). The highest performance of the 10 best performers have also been gathered every year in Track and Field resulting in 36861 data points. It will be referred to as “*10 best*” (*i.e.* regrouping the personal best value established by each of the 10 best performers every year).

### Variables

Sixty three nations possess WR. In our analysis:

- Russia represents Russia before and after soviet era, USSR, EUN (at the OG 1992, some countries of the ex-USSR competed in the EUN team (Equipe Unifiée) for the Commonwealth of Independent States),
- Czechoslovakia represents Czechoslovakia, Czech Republic and Slovakia,
- Germany represents Federal Republic German and unified Germany,
- GDR represents German Democratic Republic.

Nations are distributed in geographical world regions (North America: USA with 528 WR and Canada with 29; Western Europe with 676; Eastern Europe with 361) based on the International Olympic Committee classification. The distribution of European countries is adjusted to history. After 1945, Bulgaria, Romania, Hungary, Poland, Czechoslovakia, Yugoslavia and East-Germany are considered in Eastern Europe. After 1990, these countries join Western Europe, whereas Belarus and Kazakhstan remain in Eastern Europe. We then identify 11 regions: North America, Western Europe, Russia, Eastern Europe, Oceania, China, North Pacific, Africa, Asia, Caribbean and South America (Table S1), according to Andreff et al. [4].

### WR and Olympic Medals

WR numbers are measured for each region, and compared to Olympic Medal numbers.

### Geographical Analysis

Usually, the barycenter is defined as the average of several points, weighted by specific coefficient. Here, the points are the geographical coordinates of the country capital; and the weighting coefficients are the WR number of this country.

The barycenter 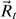 of the WR geographical coordinates (latitude, longitude) is calculated yearly, and defined as:
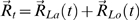



For the year *t*, 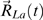 the latitudes barycenter and 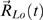 the longitudes barycenter are defined as:
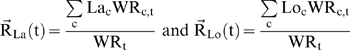

*c* is the country, *La_c_* the latitude of the capital city of the country *c*, and *Lo_c_* the longitude of the capital city of the country *c*. 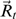 defines the path of the WR barycenter throughout the Olympic era.

### Home WR

If a performer establishes a new WR in his own country - *i.e.* WR performer citizenship and WR location coincide - the WR is defined as a “Home WR”.

Two indicators are introduced in order to further describe Home WR.

- Factor *H_t,_* the annual ratio of Home WR over the total WR number per year *t*:
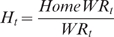
with *HomeWR_ t_* the total of WR established “at home” for the year *t*, and *WR_t_* the total of WR for the year *t*.
- Factor *H_C,_* the ratio over the whole Olympic era of Home WR number of the country *c* (*HomeWR_c_*) over the total of WR established in the host country *c* (*HostWR_c_*):
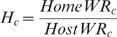

Thereafter, *H_C_* will be defined as “the rate of return” of a country organizing competitions.

### Historical Analysis

The cumulative proportions are used to describe WR secular evolution.

- Factor *a_t_* is the annual ratio of the number of WR over the total number of WR:
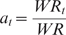

- *G* is the annual cumulative proportion over the Olympic era:
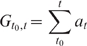
for the first year *t_0_*, until year *t*. Factor *G* defines the global (all regions) annual cumulative rate of progression of WR.
- Factor *a_r,t_* is the annual ratio of the number of WR for the region *r* over the total number of WR:
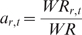

- *P* is the annual cumulative proportion over the Olympic era:
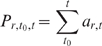
for the first year *t_0_*, until year *t* and the region *r*. Factor *P* defines the annual cumulative WR progression rate for each region.

For the evolution analysis of factors *P* and *G*, the mean slope of annual cumulative proportions (*S*) is calculated by linear regressions over 3 periods: 1918–1949, 1950–1989, and 1990–2008. S is defined as:
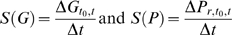
We will notify *S*_1_ = *S*_1918–1939_, *S*_2_ = *S*_1950–1989_ (*S*_2a_ = *S*_1950–1969_, *S*_2b_ = *S*_1970–1989_), and *S*_3_ = *S*_1990–2008_.

The cumulative proportions for the *10 best* have been similarly calculated (Figure S1).

Statistical analysis is performed with the R software [11]. One way linear regressions were used to calculate for the slope of factor G and P. Statistical significance was considered at p<0.05.

## Results

### WR and Olympic Medals

**Figure 1:**
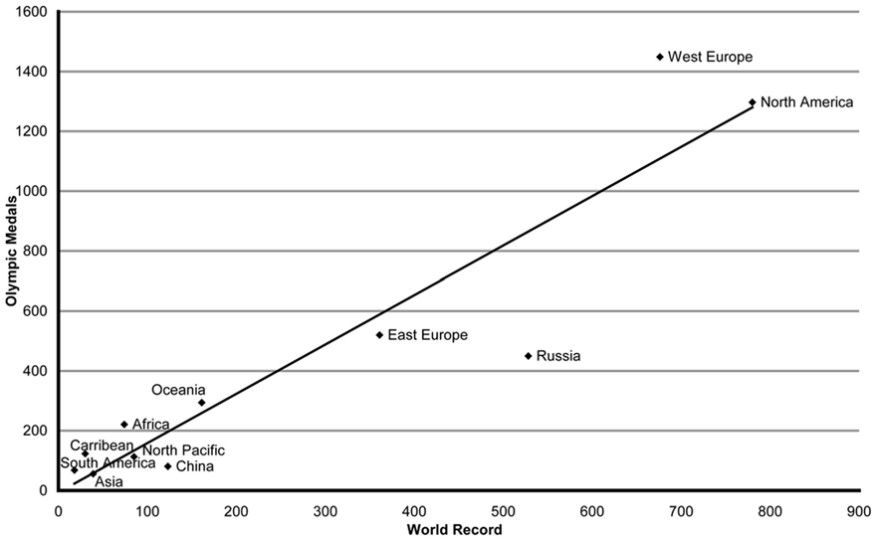
Relation between WR and Olympic medals : the correlation is established by world regions between the total number of WR and medals (Linear model: y = 1.65+6.35, F (1, 9) = 58.33, p<0.001). The correlation coefficient is 0.93.

By region, WR number is linearly related to Olympic Medals number (Figure 1).

### Geographical Analysis

**Figure 2:**
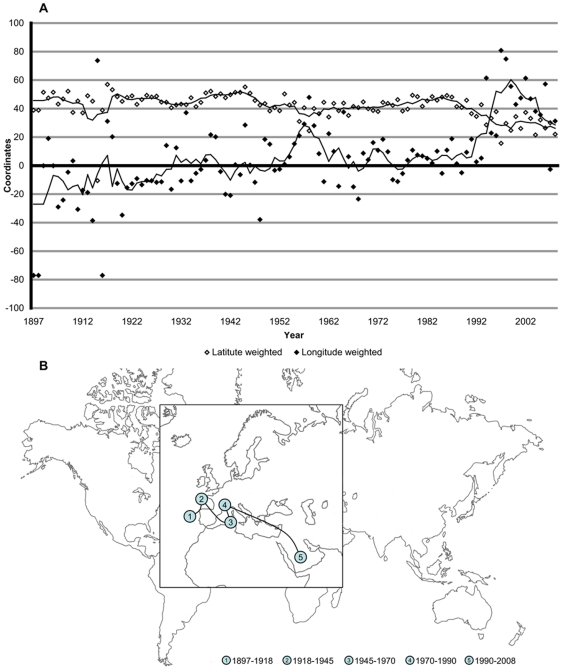
Geographical analysis of WR. A. Yearly longitude and latitude of the WR from 1897 to 2008. Coordinates are angular measurement in degree. B. The path of the barycenter of WR from 1897 to 2008. For 5 historical periods, the average barycenter is calculated: 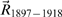
(42°31′, 14°31′), 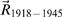
(47°26′, 2°53′), 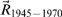
(41°13′, 7°15′), 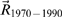
(44°01′, 5°09′), 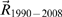
(31°10′, 37°17′), respectively (latitude, longitude).

The latitude of the WR barycenter (Figure 2A) has low variations until 1986 (mean: 43°47′+/− 8°7′). It decreases until 1999, and remains quasi-constant (mean: 28°34′+/− 5°33′) since then. The longitude grows and reaches two East peak, one in 1957 and another in 1999. The coordinates of the barycenter for the first four periods are located in Western Europe, and then moves toward South-East (13°50′ toward South, 33°46′ toward East) between 1990 and 2008 (Figure 2B).

### Regional and National Analysis

**Figure 3:**
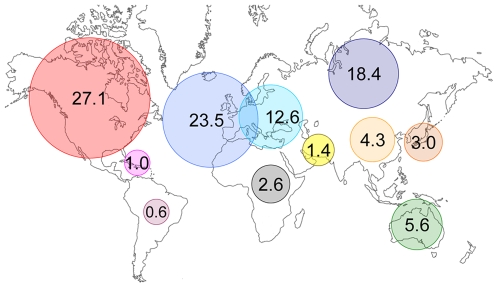
Proportion of WR by world region. North America: 27.1%, Western Europe: 23.5%, Russia: 18.4%, Eastern Europe: 12.6%, Oceania: 5.6%, China: 4.3%, North Pacific: 3.0%, Africa: 2.6%, Asia: 1.4%, Caribbean: 1.0%, South America: 0.6%.

As for Olympic medals, the major two regions that hold WR are North America (26.1%, 27.8%, respectively for WR and Olympic Medals) and Western Europe (23.5%, 30.6%) (Figure 3). Nations rank in the WR list and medal list are: USA (26.1%, 25.8% respectively), Russia (18.4%, 9.6%), GDR (6.1%, 5.4%) and Germany (5.7%, 5.8%).

### Analysis of Home WR

**Figure 4:**
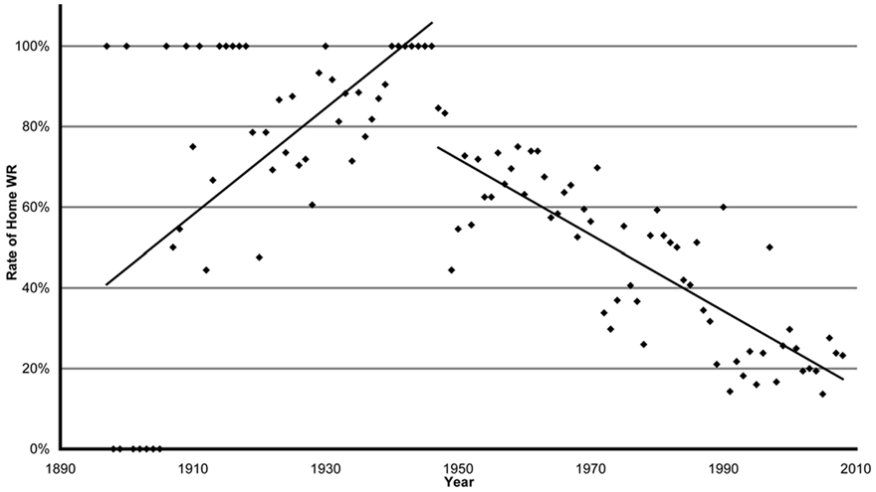
Evolution of factor *H_t_* from 1897 to 2008: yearly, Home WR over the WR total. *H_t_* increases from 1897 to 1946 (Linear Model: y = 0.013x−24.71, F (1, 48) = 23.53, p = 0.01), and decreases since 1947 (Linear Model y = −0.0095x+19.18, F (1, 60) = 143 p<0.001).

The percentage of WR established “at home” is 54.9%. Evolution of national factor *H_t_* reveals two phases (Figure 4). The first one from 1897 to 1946 shows increasing values (slope = 0.0132, mean = 79.88%), the second from 1947 (H_1947_ = 84.6%) to 2008 (H_2008_ = 23.3%) shows decreasing values (slope = −0.0094, mean = 47.63%). Between 1990 and 2008, factor *H_t_* has stabilized at 23.14%.

The rate of return for countries organizing competitions (H_C_) is 88.3% (361/409) for Russia, 85.6% (458/535) for USA, and 71.1% (54/76) for Eastern Germany (Table 1).

**Table 1:** Rate of return of Home WR (H_C_) of a country organizing a competition.

	WR beaten in the country 1897–2008 (number)	Home WR number (%)
Russia	409	361 (88.3)
USA	535	458 (85.6)
GDR	76	54 (71.1)
Nederland	64	39 (60.9)
Hungary	51	30 (58.8)
China	72	41 (56.9)
Bulgaria	41	23 (56.1)
Poland	42	23 (54.8)
Australia	142	77 (54.2)
Denmark	29	15 (51.7)
Great Britain	66	34 (51.5)
Czechoslovakia	43	22 (51.2)
France	88	45 (51.1)
Austria	44	22 (50.0)
Germany	261	120 (46.0)
Japan	123	50 (40.7)
Finland	62	25 (40.3)
Sweden	78	26 (33.3)
Italia	76	16 (21.1)
Canada	75	11 (14.7)

H_C_ of countries with a WR total above 20. Data are classified by decreasing order of percentages of Home WR beaten.

### Historical Analysis

**Figure 5:**
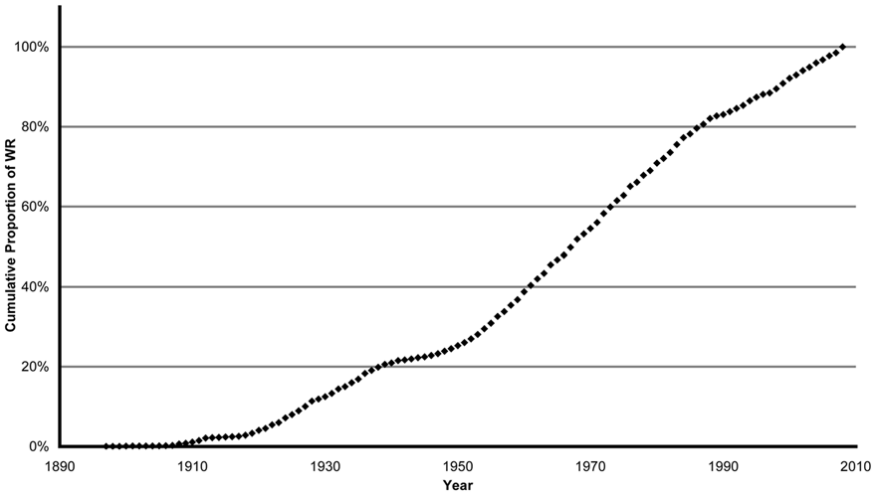
Evolution of factor G: Global (all regions) Annual Cumulative Proportions of WR (Linear Model: y = 0.0101x−19.32, F (1,110) = 2530, p<0.001).

Factor G progresses in 5 successive periods. The progression is slow for the 1897–1918 and 1939–1950 periods. Progression is similar in 1918–1939 and 1990–2008 eras, (*S*_1_ = 0.0087, *S*_3_ = 0.0094) and steeper during the 1950–1990 period, (*S*_2_ = 0.0155) (Figure 5, Table 2).

**Table 2:** Global slopes (*S*(G) for all regions) and periodic slopes (*S*(P*_r_*) for every region).

Region	Year of First WR	Slope Global	Slopes by periods			
		**1897–2008**	**1918–1949**	**1950–1989**		**1990–2008**
**Global**	**1897**	0.0101	0.0075	0.0156		0.0094
**North America**	**1897**	0.0029	0.0021	0.0046		0.0015
**Western Europe**	**1901**	0.0023	0.0040	0.0018		0.0022
**Russia**	**1917**	0.0025	0.0006	0.0038		0.0008
				**1950–1969**	**1970–1989**	
**Eastern Europe**	**1946**	0.0026	0.0003	0.0016	0.0045	0.0003
**China**	**1956**	0.0007		0.0002		0.0020
**Oceania**	**1910**	0.0006	0.0001	0.0008		0.0011
**Africa**	**1928**	0.0002	0.0002	0.0002		0.0007

p<0.001 for all results except Eastern Europe 1918–1949 (p = 0.087).

### Regional Historical Analysis

**Figure 6:**
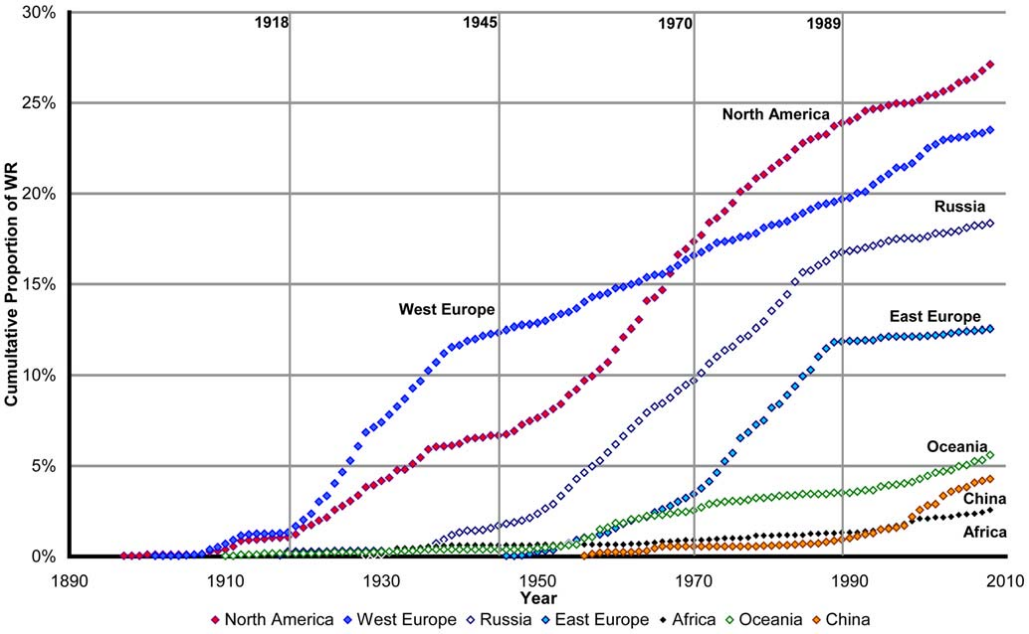
Evolution of factor P: Annual Cumulative Proportions of WR by region. P is calculated for 7 regions: North America, Western Europe, Russia, Eastern Europe, Oceania, China and Africa.

The progression of Western Europe during the second period (1950–1989) is lower (S_2_ = 0.0018) than that of the first period (1918–1949: S_1_ = 0.0040). During the second period, the progressions of USA (S_2_ = 0.0046), Russia (S_2_ = 0.0038) and Eastern Europe (S_2_ = 0.0045) are almost parallel. During the last period (1990–2008), the rate of Russian and Eastern Europe progression decreases (S_3_ = 0.0008 and S_3_ = 0.0003 respectively); whereas China's progression accelerates (S_3_ = 0.0020) (Figure 6).

The global and periodic slopes for every region are described in table 2.

A similar analysis of the 10 best performers each year shows parallel progressions for Western and Eastern Europe, North America and Russia from 1950 to 2008 (Figure S1).

## Discussion

Through a regional analysis of WR evolution during the Olympic era, this study underlines the positive or negative impact of historical (World Wars I and II, Cold War), political (Cultural Revolution), economical (Great Depression, Emergence of China) and sports factors (OG organisation, swimsuits introduced in 1999) on human performance. The WR analysis through a national prism proposes a new measurement tool of the history of human physiology during the 20^th^ and 21^st^ century. Bernard and Busse [3] have shown the link between economy and sports performances, reflecting a reading of political and economical rivalries: phases of development in the different world regions also match the historical period described here.

Our calculation of weighted annual barycenters is both a geographical and historical indicator. The average barycenters (Figure 2B) are located in Europe until 1990, then shifts towards south-east. The highest percentage of WR is European (23.5% West Europe, 12.6% East Europe, 18.4% Russia) (Figure 3) and most of OG are organised in this region (15 of 26 summer OG) [12]. Moreover, the path of WR barycenter follows the context of economical development for Western Europe, USA and China. Europe is the first world sport pole during the Olympic era. On the other hand, five out of the 63 nations holding at least one WR established more than 100 of them: USA (751), Russia (528), GDR (339), Germany (174), Australia (155) and China (123).

Others studies have established the link between Olympic medal number of a country and its political, cultural or economical development [3], [4]. The novelty is to evaluate here the link between national strategies and the evolution of WR. Moreover, the study of WR allows for precisely measuring the performance gaps between countries, and the Home WR variable (42 host nations for WR versus 16 only for OG).

### Home WR

Until 1946, the majority of WR were established “at home” (Figure 4). The geographical distance or the 1929 crisis made travels difficult and expensive for non-European countries [12]. After World War II, the Home WR rate decreased from 79.9% to 23.3% in 2008, with the expansion of travels and the participation of a growing number of athletes and nations. Since 1990, this rate stagnates: the new regions involved in sport may not have the infrastructures to host major international competitions [12]. The change of the barycenter for the last period shows the actual globalization with Africa and China appearing in the WR archives.

USA, Russia and GDR organised a large part of international competitions and had a particularly high rate of return for Home WR. Several hypotheses can be raised: the motivation of the athlete hoping for greater recognition, public support, the lack of jet lag or cultural lag [13], the establishment of a policy of return (government pressure on the athletes [14], [15]) or more conciliatory checks of the validity of performances and lighter anti-doping procedures.

### Global Curve

The slope of the global annual cumulative proportions stagnates between 1912 and 1918, then increases until 1949 (*S_1_(G)* = 0.0075) (Figure 5). While it almost doubles between 1950 and 1989 (*S_2_(G)* = 0.0156), the Cold War period starts with the Truman's doctrine formulation in 1947 [16] and the Marshall plan for West-European and North American countries, followed by the creation of the Cominform and the formulation of Zhdanov's doctrine for Russia and East-Europe [17], [18]. The slope of progression for the 1990–2008 era decelerates (*S_3_(G)* = 0.0094) as WR rarefy [1]. A last and small increase appears in 1999 with the first women record in weightlifting [11], and the new records in swimming partially due to the first generation swimsuits [19]–[21]. The deceleration of WR progression after 1988 follows the new geopolitics of the 1990 (Fall of Berlin Wall and USSR end). It is also the end of an exacerbated period of competition motivated by Cold War.

### Western Europe

Western Europe is the first sport region until 1939. After the Second World War, Western Europe has to rebuild its territory and economy [16], [17]: its annual cumulative proportion slows. Analysis of the 10 best performers (Figure S1) shows a paradox: although constantly present after 1940 among the top 10, it does not receive the dividends of this investment as WR. Western Europe does not seem to be as involved as USA and Soviet Union in confrontations for the first places, a high field opposition between the two blocs. With the new geopolitical distribution after 1990, its slope of progress slightly reincreases, and yields new record rates due to swimming performances after 1999 [19]–[21].

### USA

First competitions until World War II were essentially hard-fought between Europe and North America. After the Great Depression, the Second World War and the Cold War have stimulated American economy [16]. In 1947, the growth of WR accelerates. USA obtains the highest slope during the Cold War, *S_2_(P_North America_)* = 0.0046. After 1989 and despite swimsuits, the slope of curve falls: *S_3_(P_North America_)* = 0.0015. USA performances may have reached their maximum in the early 70s.

### Russia

Russia effectively begins its WR series in 1935 (Figure 6). This period corresponds to the beginning of a massive industrial investment wanted by Stalin in the five-year plans, and to the Great Purges of the Communist Party and first Moscow Trials [17]. In 1947, Russia formulates the Zhdanov's doctrine [18]. The Russian curve increases again in 1950. In 1952, for the first time since the 1917 revolution, the Soviet Union participates to the Helsinki OG. Between 1950 and 1989, Russian growth is the highest (*S_2_(P_Russia_)* = 0.0038) though weaker than the US one. In 1983, the Russian curve shows a slowdown at the time of Perestroika [17] and Gorbachev's Glasnost. After 1990, the progression rate of Russian WR declines to *S_3_(P_Russia_)* = 0.0008 (4.7 times lower). The Warsaw Pact dissolved; Russia enters a transition phase. The growth of Russians performances reached its maximum early in the 80s and stagnates in the 90s. This reminds the evolution of Russian life expectancy that no longer progressed after 1975 [22].

### Eastern Europe

USSR pace is followed by popular democracies in Eastern Europe [12]. The first record of the region dates back to 1946. Until 1970, its progression is low, *S_2a_(P_Eastern Europe_)* = 0.0016. Between 1970 and 1989, the curve increases sharply, *S_2b_(P_Eastern Europe_)* = 0.0045. East Germany holds 48% of Eastern European records and presents the greatest slope of this region as GDR Communist Party looks for worldwide recognition [12], [14]. Until Germany's reunification, GDR athletes dominate sports competitions. Thereafter, the Stasi archives showed a sport organization with early and methodical detecting systems and institutionalized State doping [12], [14]–[15], [23]. After German reunification, the former countries of Eastern Europe reorganise their system and greatly reduced the budget share devoted to sports [24].

### China

The first Chinese record dates back from 1956. At that time, Mao Zedong declares “Sport upholds the dignity and independence of the Chinese nation” [25]. The Chinese government introduces the “Ten-year Guidelines Sports development” [25]. Then the progression of Chinese WR is brutally stopped in 1966, when China begins its Cultural Revolution. For 10 years the development of high-performance level is interrupted. Three years after the death of Mao Zedong, China breaks a new WR, while his government changes policy [25]. After its participation to the 1984 OG, decision is taken to accelerate the reforms of the sport system and to invest in high-level sports and organization of international competitions [12], [25]. In 1986, the Chinese curve increases its slope of progression. During the period of maximal economic development (1990–2008), the slope of growth of Chinese WR accelerates (at a 10 times higher pace, *S_3_(P_China_) = 0.0020)*, while Beijing becomes candidate to host the OG [12]. Many German and Russian coaches are recruited [26]. Chinese WR include a major representation of women performances (weightlifting female champions hold 50.4% of all Chinese WR). The Chinese WR curve increases during the 5^th^ period, when other regions slow down, starting to reach physiological limits [1], [2].

### Oceania and Africa

Oceania curve (Australia: 155 WR, New Zealand: 6 WR) really starts in 1956, with the first Oceania's OG in Melbourne, and increases again with the Sydney OG (2000) and the numerous swimming records [19]–[21].

Early African WR were due to Egyptian weighlifters but 50% of them come from present East African runners (Kenya: 19, Ethiopia: 14, Tanzania: 1, Uganda: 1). Africa is mainly represented in the last period with a 3.8 fold slope increase.

### Cold War

During Cold War, sports competitions like other areas (Space and Moon race, Nuclear arms race) become places of ideological confrontation [16], [26]. US candidatures to organize OG have multiplied [12]. From 1970 to 1989, the ACP slopes of Russia, East-Europe and North America are almost parallel. The doping state of GDR athletes is now known and published [14]–[15], [23]. That of Soviet athletes is recognized by athletes and coaches from the former USSR [15], [27]. All of them compete against American athletes with the following progression slope: *S*_2_(P_Eastern Europe_) = 0.0031; *S*_2_(P_Russia_) = 0.0038; *S_2_*(P_USA_) = 0.0046. In 1988, Soviet and American athletes met one last time at the Seoul OG, after a 12 year break. Seoul OG resulted in 2 Soviet records, 5 East-European and 9 American records for a total of 19 versus 12 in Los Angeles (1984) and 13 in Barcelona (1992); this shows the particular intensity of this last Cold War competition that reached an unprecedented peak of atypical performances including the everlasting sprint world records by Florence Griffith-Joyner [28].

### Limitations

In order to have international comparison, we only took the four quantifiable summer disciplines, as two countries only in southern hemisphere (Australia and New-Zealand) have WR in speed skating [29]. We also adjusted the country distribution according to the geopolitics of 1945 and that of 1990. This deprives of a strict secular follow-up of East-European countries, but the choice is relevant for a focus on the Cold War geopolitical interactions.

### Conclusion

This analysis proposes new indicators of human performances throughout the Olympic era. Annual cumulative proportions of WR highlight economical, geopolitical and developmental challenges around sport during the 20^th^ and 21^st^ centuries. Detaining a record allows the athlete, his nation and sponsors to win world recognition. Regions of the world that hold a significant number of them have been political leaders or strong emerging economic forces [12]. The measure of WR progresses provides quantifiable indicators to follow-up historical and geostrategic decisions. Sport may therefore be an interesting tool for measuring development. Such a vision might benefit from upcoming models integrating broader parameters such as gross domestic product, life expectancy or demography.

## Supporting Information

Figure S1 Annual Cumulative Proportions of 10 best by region for Track and Field. P is calculated for 8 regions: North America, Western Europe, Russia, Eastern Europe, Oceania, China, Africa and Carribean.
(0.33 MB TIF)

Table S1 Distribution of countries according to regions and periods. NAm: North America, SAm: South America, Car: Carribean, Afr: Africa, WEu: Western Europe, EEu: Eastern Europe, Rus: Russia, Asi: Asia, Chi: China, NPa: North Pacific, Oce: Oceania.
(0.03 MB XLS)
