# Supplementary figures and images for: Success in Developing Regions: World Records Evolution through a Geopolitical Prism
Source: PLoS One. 2009 Oct 28;4(10):e7573. doi: 10.1371/journal.pone.0007573 (PMC2763220; doi:10.1371/journal.pone.0007573)

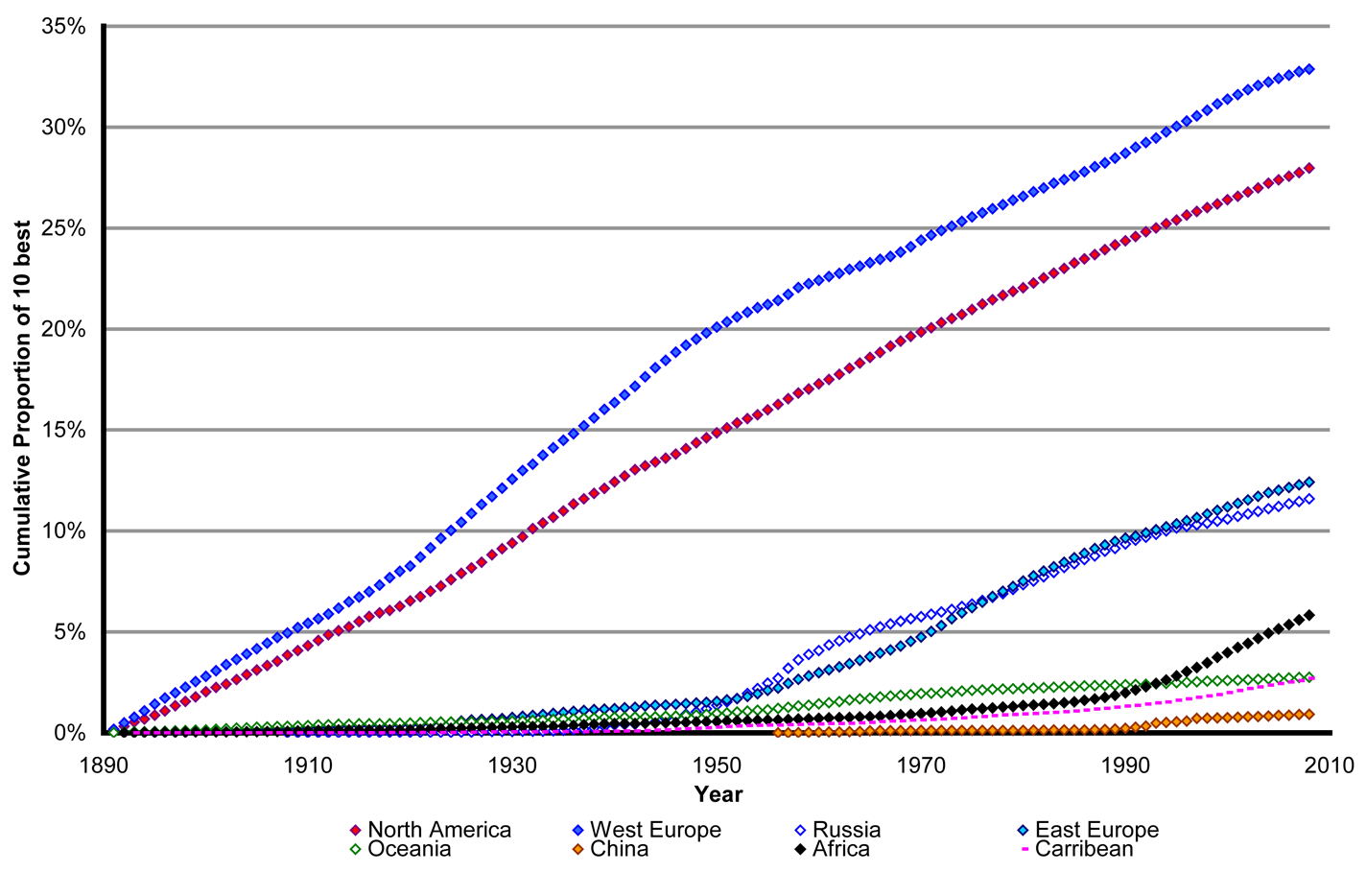

Supplement: Figure S1 — Annual Cumulative Proportions of 10 best by region for Track and Field. P is calculated for 8 regions: North America, Western Europe, Russia, Eastern Europe, Oceania, China, Africa and Carribean. (0.33 MB TIF) [file pone.0007573.s001.tif]
